# An 11-month-old boy with tuberculous meningitis presenting as progressive limb weakness, fever, developmental retardation, and loss of consciousness: a case report

**DOI:** 10.1186/s13256-024-04523-1

**Published:** 2024-04-27

**Authors:** Guive Sharifi, Mohammad Ansari, Elmira Mahmoudi Chalmiani, Farid Javandoust Gharehbagh, Ilad Alavi Darazam

**Affiliations:** 1 Skull Base Research Center, Loghman Hakim Hospital, Shahid Beheshti University of Medical Sciences, Tehran, Iran; 2 Infectious Diseases and Tropical Medicine Research Center, Shahid Beheshti University of Medical Sciences, Tehran, Iran; 3 Department of Infectious Diseases and Tropical Medicine, Loghman Hakim Hospital, Shahid Beheshti University of Medical Sciences, Makhsoos St, South Kargar Ave, Tehran, Iran

**Keywords:** Mycobacterium tuberculosis, Meningitis, Hydrocephalus, Developmental retardation, Fever, Limb weakness, Loss of consciousness, Case report

## Abstract

**Background:**

Tuberculous meningitis (TBM) accounts for about 1% of all tuberculosis cases and about 5% of extrapulmonary tuberculosis cases. However, it poses major importance because approximately half of those affected die or become severely disabled. Herein, the successful treatment of an 11-month-old boy with progressive limb weakness, fever, developmental retardation, and loss of consciousness due to tuberculosis, was reported.

**Case presentation:**

An 11-month-old (Iranian Turk) boy was referred to Loghman Hakim hospital for progressive limb weakness and loss of previously attained developmental milestones for the past 2 months. He also had persistent fever and loss of consciousness for about 14 to 21 days. Before being referred to our center, the patient had been diagnosed with hydrocephalus at another center due to possible acute bacterial meningitis based on a CT scan and MRI imaging. On physical examination, anterior fontanel bulging and neck stiffness were observed on the admission. His body temperature and heart rate were 38.1 C and 86 beats per minute (bpm), respectively. He had left 6 cranial nerve palsy and spastic quadriparesis with a power of grade 3/5. Other systemic examinations were normal. Endoscopic third ventriculostomy (ETV) (and leptomeningeal biopsy) revealed diffuse thickening of the floor and lateral walls of the 3rd ventricle and also a cobblestone appearance in the form of multiple white patchy lesions was detected on the floor of the 3rd ventricle. CSF analysis and polymerase chain reaction confirmed the TB meningitis. During hospitalization, a temporary EVD (external ventricular drain) was initially inserted. Eventually, defervescence was denoted 5–6 days after initiation of anti-TB medications, and a permanent ventriculoperitoneal shunt was inserted due to hydrocephalus. Gradually his truncal and limb tone and motor function improved, as did his emotional responses to his parents and ability to eat. The patient can walk without help in the 15th month following the operation and resolved hydrocephalus demonstrated on follow-up imaging.

**Conclusion:**

Over half of treated TB meningitis patients die or suffer severe neurological sequelae, mainly due to late diagnosis. Hence, early diagnosis and prompt initiation of TB treatment offer the best chance of a good neurological outcome.

**Supplementary Information:**

The online version contains supplementary material available at 10.1186/s13256-024-04523-1.

## Introduction

The tuberculosis is a typical reason for the meningitis in the developing countries which are characterized by the high number of pulmonary tuberculosis cases [1]. In 2017, approximately 10 million people had tuberculosis worldwide, with more than 100,000 new cases of tuberculous meningitis (TBM) estimated to occur each year [2]. Tuberculous meningitis is responsible for 20–60% of TB- related deaths in children [3]. In Iran, most tuberculous meningitis cases in children were reported in the age range from 6 months to 4 years, with an incidence of 31.5 per 100,000 in this age group and 0.7 per 100,000 in older children (10–14 years) [4] TBM is the most destructive and progressive form of TB. It is expressed as the only manifestation of TB, or can coexist with the other sites such as lung (pulmonary) or extrapulmonary sites of infection. TBM causes the usual range of the symptoms and indications of meningitis, headache, fever, and neck stiffness, (however), meningeal signs may disappear in its early stages. The length of time taken for the signs and symptoms to show up has a range of few days to several months [1]. In Iranian research, the most prevalent symptoms of tuberculous meningitis were fever and anorexia, headache and stiff neck (90%), decreased level of consciousness and vomiting (80%), seizure (50%), cranial nerve palsy (30–50%), and elevated intracranial pressure (40%). The course of TB meningitis is slow, with an aseptic pattern in the early stages. A delay in diagnosis can lead to irreversible and severe neurologic consequences [5]. Gadolinium-enhanced MRI enables visualization of leptomeningeal tubercles in approximately 90% of children and 70% of affected adults. The diagnosis of TBM is often delayed due to the poor sensitivity of culture techniques and the time required to confirm the disease [2]. Vascular infarction is the mechanism responsible for many diverse clinical neurological abnormalities in TBM patients and accounts for a significant portion of irreversible neurological sequelae [6]. Early diagnosis and prompt initiation of TB treatment offer the best chance for a favorable neurological outcome. Without appropriate treatment, the disease will inevitably progress, leading to neurological deficits, CNS damage, and, in many cases, death. More than half of treated TBM patients die or suffer severe neurological sequelae, mainly due to late diagnosis [2].

## Case presentation

An 11-month-old Iranian Turk boy was referred to Loghman Hakim Hospital for progressive limb weakness and loss of previously attained developmental milestones for the past 2 months. He also had persistent fever and loss of consciousness for about 14 to 21 days (please write how many days). He was born at 35 weeks of gestation by cesarean section with normal birth weight (2650 g), length (50 cm), and head circumference (35 cm). He received BCG and other routine immunizations. His neonatal period, development up to 9 months, and family history were unremarkable. His parents noticed progressive weakness of all four limbs and loss of head control along with the inability to creep or crawl, at 11 months of age. He would constantly cry and be irritable. Subsequently, he was unresponsiveness and lost consciousness. For these complaints, he was taken to a hospital where he had been diagnosed with hydrocephalus possibly due to acute bacterial meningitis based on a CT scan and MRI imaging of the brain. He was treated with acetazolamide, ceftriaxone, corticosteroid, and antipyretic for 11 days.

**Fig. 1:**
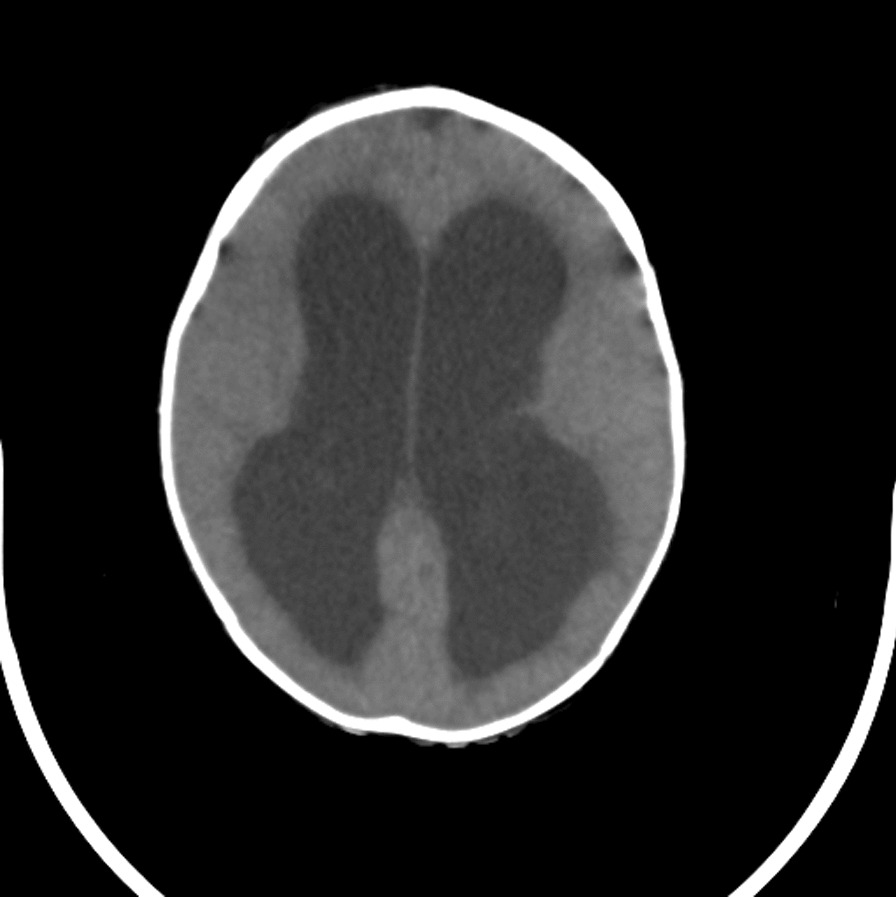
Brain Computerized tomography scan without contrast injection. Hydrocephalus and elevated intracranial pressure

**Fig. 2:**
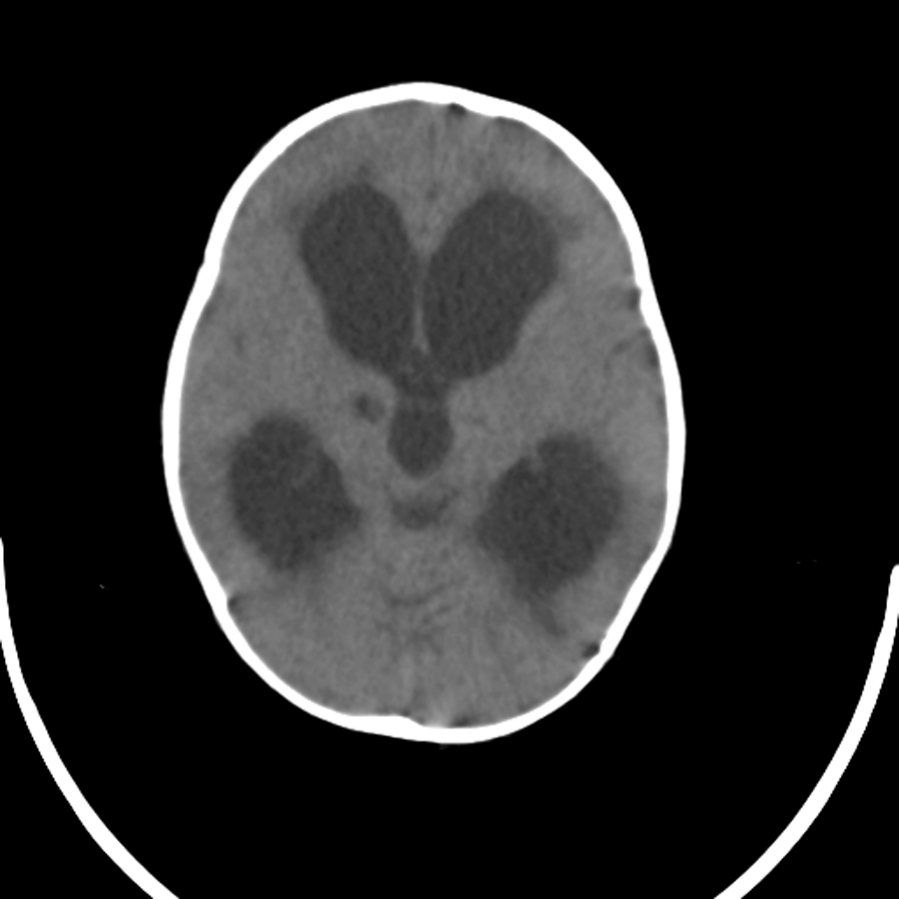
Brain Computerized tomography scan without contrast injection. Hydrocephalus, elevated intracranial pressure and hypodensity consistent with infarct-induced ischemia at right thalamus

**Fig. 3:**
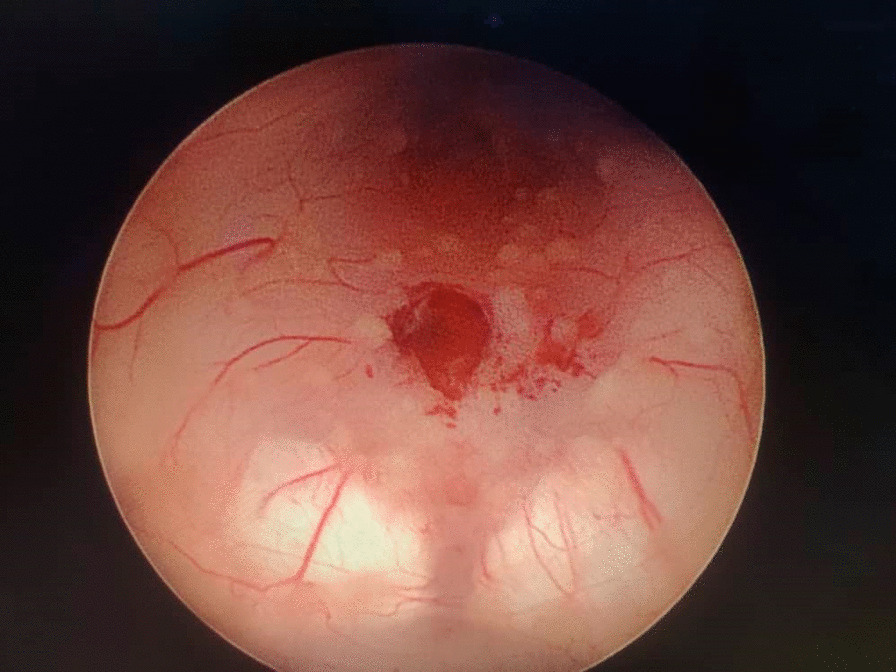
Multiple white patchy lesions seen at the floor of the 3rd ventricle within the tuber cinereum during Endoscopic third ventriculostomy

**Fig. 4:**
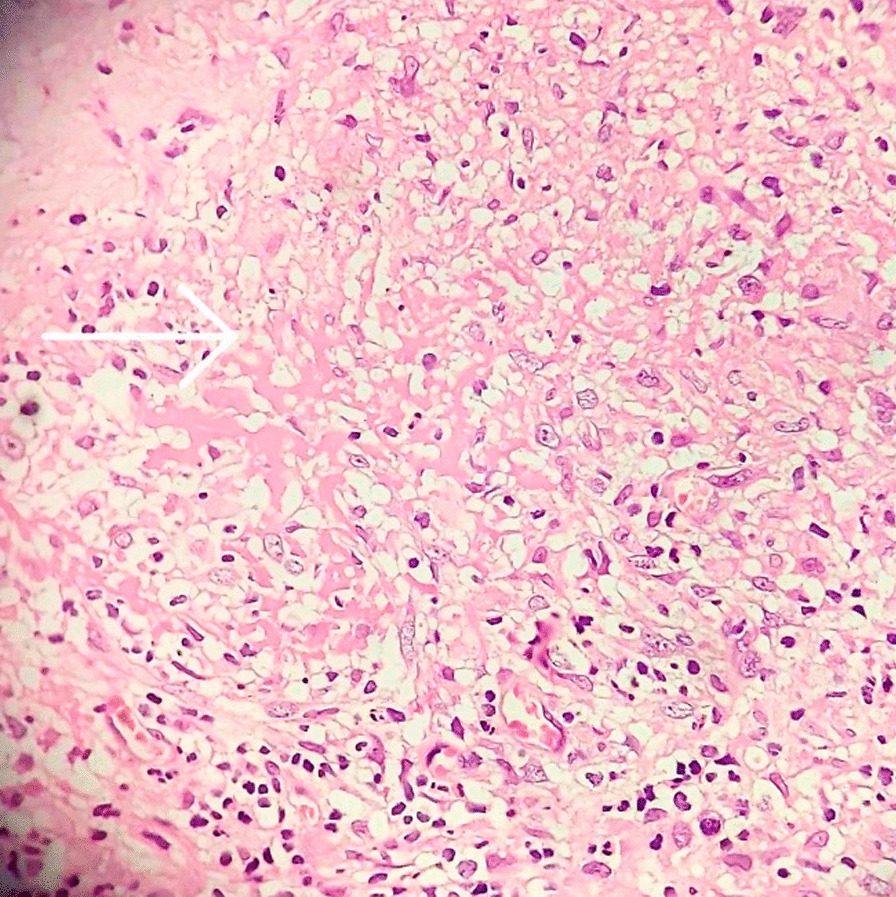
Foreign body types multinucleated giant cells and areas of acellular necrosis (indicated by arrow). Acid-fast bacilli are not seen with Ziehl–Neelsen staining

**Fig. 5:**
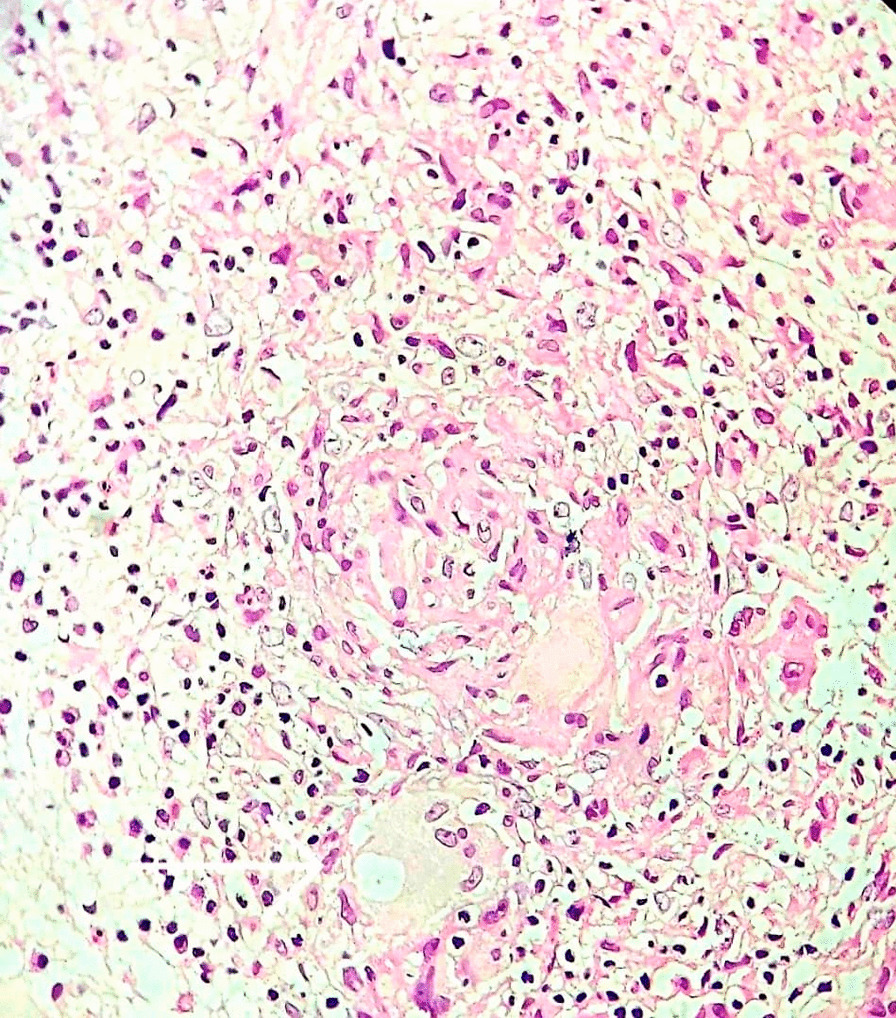
Multiple well-demarcated granulomas composed of epithelioid histiocytes Langerhans cells

The patient was referred to our hospital due to clinical deterioration and the onset of new symptoms. Additionally, his parents noted convergence deviation of the eyes and flexion of the lower limbs when held upright. He was ill, febrile (38.6 degrees Celsius axillary), lethargic, and continuously crying. He lost head control and was unable to crawl which he was doing earlier. On physical examination at our center, the anterior fontanel was bulging and there was neck stiffness. Left 6th cranial nerve paresis and quadriparesis (3/5 for both upper and lower limbs) was present. Hyperreflexia (DTR 3/4) and extensor plantar reflexes bilaterally were observed. Right basal ganglia hypodensity consistent with infarct-induced ischemia along with four ventricular hydrocephalus could be noticed in brain CT scans without contrast injection (Figs. 1 and 2). Brain MRI revealed severe four-ventricular hydrocephalus with peri-ependymal edema associated with significant leptomeningeal enhancement of the intracranial cisterns and sulci (see Additional files 1 and 2). There were some enhancing foci at bilateral thalami and pons. Based on hydrocephalus, basal ganglia infarction, and the leptomeningeal enhancement, it was decided to perform endoscopic third ventriculostomy (ETV) (and leptomeningeal biopsy) as the diagnostic and therapeutic approaches. During ETV, diffuse thickening of the floor and lateral walls of the 3rd ventricle and also a cobblestone appearance in the form of multiple white patchy lesions was detected on the floor of the 3rd ventricle (Fig. 3). CSF obtained during ETV suggested TB meningitis (protein: 183 mg/dL (normal range: 15–45 mg/dl), glucose 56 mg/dL (normal range: 45–80 mg/dl), blood glucose: 109 mg/dL (normal range: 70–179 mg/dl), WBC: 55/mm3 (normal range: 0–25/mm3), RBC: 0/mm3 (normal range: 0–5/mm3), PMN: 10%, MN: 90%); Therefore, empiric three-drug regimen with isoniazid, pyrazinamide, and rifampin was started. Finally, CSF analysis revealed acid-fast bacilli in Ziehl–Neelsen staining, and Mycobacterium tuberculosis was detected by polymerase chain reaction (PCR). The histopathologic examination revealed multiple well demarcated granulomas composed of epithelioid histiocytes Langerhans cells and foreign body types multinucleated giant cells and areas of acellular necrosis (Figs. 4 and 5), confirming the diagnosis of TB.

A temporary EVD (external ventricular drain) was initially inserted. Eventually, defervescence was noted 6 days after initiation of anti-TB medications, and a permanent ventriculoperitoneal shunt was inserted. Gradually his truncal and limb tone and motor function improved, as did his emotional responses to his parents and ability to eat. Thirteen months after treatment, he was conscious and responsive to environmental stimuli. He was neither febrile nor irritable. He regained the developmental milestones, and limb strength was markedly improved (4/5 for upper and lower limbs). The child could walk without help in the 15th month following the operation and resolved hydrocephalus demonstrated on follow-up (see Additional file 3).

## Discussion

In this study, an interesting case of TBM who was initially treated as pyogenic meningitis in another center, was reported. Due to the progression of the illness and new symptoms, the patient was referred to our center where his diagnosis was confirmed with TBM with relevant investigations Tuberculous meningitis accounts for approximately 1% of all tuberculosis cases and around 5% of extrapulmonary tuberculosis cases. Despite its relative rarity, it is a significant concern as roughly half of those affected either die or become severely disabled [6]. Tuberculous meningitis is the deadliest and most disabling form of tuberculosis [7] and may be responsible for the deaths of more than 200 children per day worldwide due to TB [2]. Around 500 000 children become tuberculosis (TB) patients annually. Furthermore, EPTB is detected in up to 20–30% of cases with each year [8]. The child’s age is significantly an important factor of concern as more than 58% of pediatric tuberculosis takes place among infants who are below the age of five and even quarter of these cases reported results in extrapulmonary tuberculosis (EPTB). [9]. Infants, young children, and those with HIV infection are often affected by severe forms of tuberculosis, such as disseminated TB or tuberculous meningitis (TBM) [10]. In children, initial symptoms are nonspecific and may include cough, fever, vomiting (but not diarrhea), malaise, and weight faltering. Initial apathy or irritability can progress to meningitis, with a decreased level of consciousness and signs of increased intracranial pressure (often indicated by a bulging anterior fontanelle and abducens nerve palsy). Focal neurological signs, such as hemiplegia, may also be observed during the physical examination [6]. Pathophysiologically, adhesions can result in cranial nerve palsies (particularly affecting nerves II, III, IV, VI, VII, and VIII), constriction of the internal carotid artery leading to stroke, and obstruction of cerebrospinal fluid (CSF) flow, causing raised intracranial pressure, reduced consciousness level, and hydrocephalus [11]. Consequently, the most common findings, in descending order, are meningeal enhancement, hydrocephalus, basal exudates, infarcts, and tuberculomas [12] Infarcts occur in approximately 30% of cases, commonly affecting the internal capsule and basal ganglia, which can lead to a range of disorders from hemiparesis to movement disorders [11]. Therefore, contrast-enhanced brain CT or MRI may be helpful in supporting the diagnosis of tuberculous meningitis, as abnormalities are frequently present in the early stages [12]. In a consecutive series of 88 HIV-negative patients with definite TB meningitis diagnosed by positive CSF culture, the following median values were determined from CSF analyses: cell count (136/µL), mononuclear cell percentage (63%), protein concentration (160 mg/dL), and CSF glucose-to-blood glucose ratio (0.13) [13]. Ziehl–Neelsen staining has a very low sensitivity in cases of TBM [14]. In the Prospective observational study from the Indonesia, as reviewed from 207 TBM sufferers, only 11% of Ziehl–Neelsen staining samples were positive [13]. Mortality rates from TBM in children range from 5 to 23%. Approximately 14% to 52% of children with TBM experience post-treatment neurological sequelae. Despite treatment, the risk of mortality is 19.3%, and the chance of survival without disability is 36.7% [10]. Initiating antitubercular treatment before the onset of coma is the strongest predictor of survival in TB meningitis [6]. Antitubercular treatment before the onset of coma is the strongest predictor of survival in TB meningitis [15]. Early diagnosis and prompt initiation of TB treatment offer the best chance for a favorable neurological outcome [2].

The late diagnosis of TBM (tuberculous meningitis) is often misled by non-specific early symptoms, as they can be confused with less dangerous conditions. With symptoms being similar to that of the common viral infections or other neurological disorders, the symptoms like fever, headache, and fatigue can imitate these conditions [16]. Furthermore, given that TBM is usually slow-running and that diagnostics such as cerebrospinal fluid analysis and mycobacterial cultures are complex, delays are inevitable when trying to identify the problem. In Ref. [16, 17]. This delay may cause very serious consequences, including a higher chance of death, neurological complications, or long-term effects. Instantiation of treatment from the beginning of the process is a key component of improving the outcome in TBM patients and this necessitates the raising of awareness, expediting of diagnostic methods, and early empirical treatment in places with high rates of TBM patients (areas with high prevalence of tuberculosis) and of the patients with suspicious clinical presentation.

## Conclusion

Tuberculous meningitis is one of the most severe and lethal forms of tuberculous infection and delayed onset of the treatment due to non-specific early signs and symptoms might be fatal. TBM in early childhood could cause diffuse leptomeningeal enhancement, tuberculoma formation within CNS, basal ganglia infarct, hydrocephalus, and finally different neurologic sequelae (developmental delay or retardation, cranial nerve palsies and …) or even death. Hence, the Early onset of the treatment is of paramount importance and life-saving.

### Supplementary Information

**Additional file 1. **T1-weighted brain MRI with gadolinium-based contrast injection showing four-ventricular hydrocephalus. There were some enhancing foci at bilateral thalami.

**Additional file 2. **T1-weighted brain MRI with gadolinium-based contrast injection showing four-ventricular hydrocephalus with peri-ependymal edema associated with diffuse significant leptomeningeal enhancement of intracranial cisterns and sulci.

**Additional file 3. **Follow-up T1-weighted MRI showing complete resolution of hydrocephalus.

## Data Availability

Not applicable.

## Abbreviations

TBM: Tuberculous meningitis
TB: Tuberculosis
EPTB: Extrapulmonary tuberculosis
CNS: Central nervous system
CT: Computerized tomography
MRI: Magnetic resonance imaging
ETV: Endoscopic third ventriculostomy
CSF: Cerebrospinal fluid
EVD: External ventricular drain
PCR: Polymerase chain reaction
