# Supplementary figures and images for: An 11-month-old boy with tuberculous meningitis presenting as progressive limb weakness, fever, developmental retardation, and loss of consciousness: a case report
Source: J Med Case Rep. 2024 Apr 27;18:210. doi: 10.1186/s13256-024-04523-1 (PMC11055343; doi:10.1186/s13256-024-04523-1)

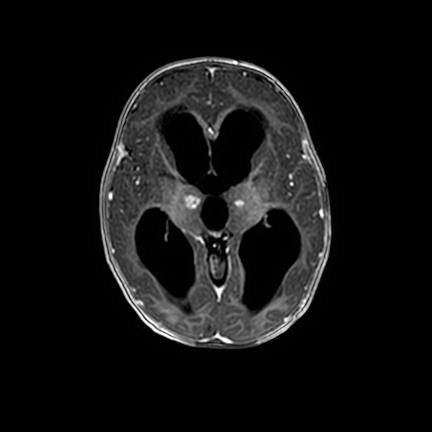

Supplement: Supplementary file 1 — Additional file 1. T1-weighted brain MRI with gadolinium-based contrast injection showing four-ventricular hydrocephalus. There were some enhancing foci at bilateral thalami. [file 13256_2024_4523_MOESM1_ESM.tif]

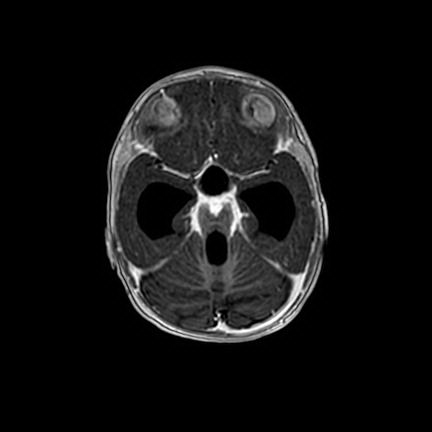

Supplement: Supplementary file 2 — Additional file 2. T1-weighted brain MRI with gadolinium-based contrast injection showing four-ventricular hydrocephalus with peri-ependymal edema associated with diffuse significant leptomeningeal enhancement of intracranial cisterns and sulci. [file 13256_2024_4523_MOESM2_ESM.tif]

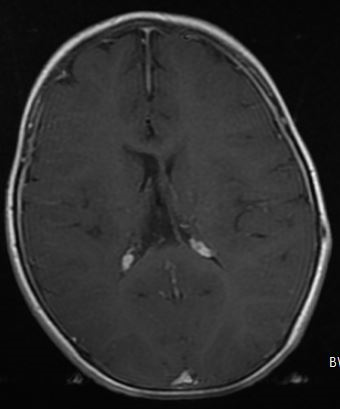

Supplement: Supplementary file 3 — Additional file 3. Follow-up T1-weighted MRI showing complete resolution of hydrocephalus. [file 13256_2024_4523_MOESM3_ESM.tif]
